# Cyanogenesis in the *Sorghum* Genus: From Genotype to Phenotype

**DOI:** 10.3390/genes13010140

**Published:** 2022-01-14

**Authors:** Max Cowan, Birger Lindberg Møller, Sally Norton, Camilla Knudsen, Christoph Crocoll, Agnelo Furtado, Robert Henry, Cecilia Blomstedt, Roslyn M. Gleadow

**Affiliations:** 1 School of Biological Sciences, Monash University, Clayton, VIC 3800, Australia; max.cowan@uq.edu.au (M.C.); cecilia.blomstedt@monash.edu (C.B.); 2 Plant Biochemistry Laboratory, Department of Plant and Environmental Sciences, University of Copenhagen, 1871 Frederiksberg, Denmark; blm@plen.ku.dk (B.L.M.); hpm627@alumni.ku.dk (C.K.); 3 Australian Grains Genebank, Agriculture Victoria, Horsham, VIC 3400, Australia; sally.norton@agriculture.vic.gov.au; 4 DynaMo Center, Section for Molecular Plant Biology, Department of Plant and Environmental Sciences, University of Copenhagen, 1871 Frederiksberg, Denmark; chcr@plen.ku.dk; 5 Queensland Alliance for Agriculture and Food Innovation, The University of Queensland, St Lucia, QLD 4072, Australia; a.furtado@uq.edu.au (A.F.); robert.henry@uq.edu.au (R.H.)

**Keywords:** *Sorghum bicolor*, wild crop relatives, dhurrin, cyanogenesis

## Abstract

Domestication has resulted in a loss of genetic diversity in our major food crops, leading to susceptibility to biotic and abiotic stresses linked with climate change. Crop wild relatives (CWR) may provide a source of novel genes potentially important for re-gaining climate resilience. *Sorghum bicolor* is an important cereal crop with wild relatives that are endemic to Australia. *Sorghum bicolor* is cyanogenic, but the cyanogenic status of wild *Sorghum* species is not well known. In this study, leaves of wild species endemic in Australia are screened for the presence of the cyanogenic glucoside dhurrin. The direct measurement of dhurrin content and the potential for dhurrin-derived HCN release (HCNp) showed that all the tested Australian wild species were essentially phenotypically acyanogenic. The unexpected low dhurrin content may reflect the variable and generally nutrient-poor environments in which they are growing in nature. Genome sequencing of six CWR and PCR amplification of the *CYP79A1* gene from additional species showed that a high conservation of key amino acids is required for correct protein function and dhurrin synthesis, pointing to the transcriptional regulation of the cyanogenic phenotype in wild sorghum as previously shown in elite sorghum.

## 1. Introduction

Sorghum (*Sorghum bicolor* (L.) Moench) is a major cereal crop and the fifth most important cereal crop worldwide. As a C_4_ plant, sorghum has several advantages over wheat and rice under harsh growing conditions as a result of increased photosynthetic efficiency and a higher tolerance to drought and elevated temperatures [1,2]. Sorghum produces the cyanogenic glucoside dhurrin in all vegetative tissues. Cyanogenesis describes the process whereby cyanogenic glucosides are hydrolyzed by specific β-glucosidases to release hydrogen cyanide (HCN) [3]. Plants avoid autotoxicity by the spatial separation of cyanogenic substrate and enzyme at the cellular or subcellular level, thus HCN is only released following tissue disruption [3,4,5]. This binary system has been demonstrated to provide plants with an immediate targeted response to herbivore attacks [6,7,8,9]. However, cyanogenesis also limits the use of sorghum as livestock feed and forage [10].

Cyanogenic glucosides are widespread throughout the plant kingdom [9,11,12,13,14,15,16,17], yet these compounds occur at a disproportionately high frequency in cultivated plants [18]. It is currently unclear why so many crop plants, including *S. bicolor,* are cyanogenic. An increased production of cyanogenic glucosides may have been indirectly selected for during domestication as a form of natural pesticide [3,18,19]. Cyanogenesis is an effective deterrent against generalist herbivores, but some of the most common and damaging insect pests of *S. bicolor* (e.g., cotton bollworm (*Helicoverpa armigera*), sorghum midge (*Stenodiplosis sorghicola*)) feed mainly on the acyanogenic mature grains, rather than the cyanogenic vegetative tissues, thereby avoiding any potential HCN toxicity effects [20,21]. The initial reasons for the domestication and early cultivation of *S. bicolor* must also be considered. Sorghum was likely cultivated primarily for the production of the acyanogenic grain, rather than as an animal feed [22,23,24,25]. The presence of dhurrin in all vegetative parts of sorghum may therefore have provided benefits in terms of deterring herbivores, without any impact of cyanide toxicity on human health.

The prevalence of cyanogenesis in domesticated and undomesticated plants has generally been studied independently [3,18]. Direct comparisons of cyanogenic traits between major crop species and their genetically isolated wild relatives have rarely been performed. For example, Nassar and Fichtner [26,27] assessed the quantitative HCN content of six undomesticated cassava (*Manihot*) species but did not examine domesticated varieties (*M. esculenta*) in the same study. Variation in cyanogenic capacity has been investigated more thoroughly in naturalized populations of domesticated species, for example in lima bean (*Phaseolous lunatus*) [9], white clover (*Trifolium repens*) [28,29], legumes (*Lotus* spp.) [30], *Macadamia* spp. [31] and the rubber tree (*Hevea* spp.) [32].

**Figure 1:**
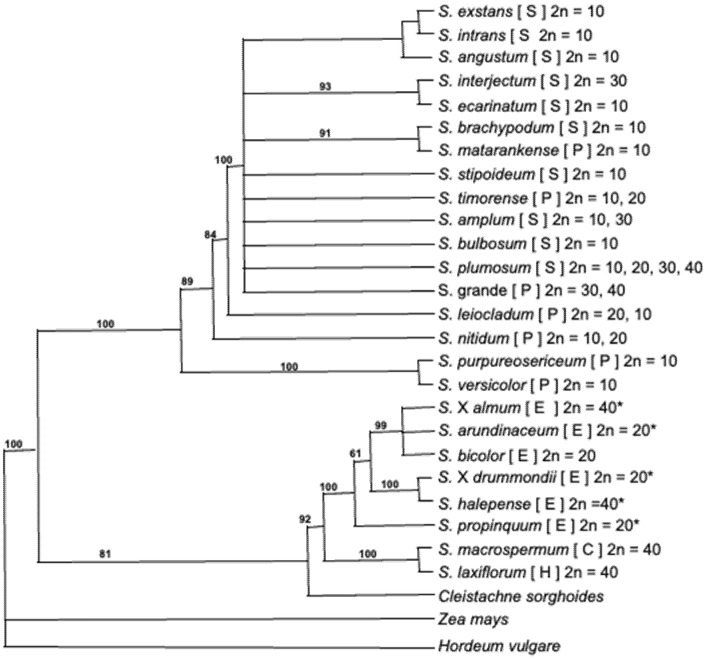
Phylogenic tree of the sorghum genus, modified from Dillon et al. [33]. Letters is parentheses after each species indicate taxonomic subgenera, where S = *Stiposorghum*, P = *Parasorghum*, E = *Eusorghum*, C = *Chaetosorghum*, H = *Heterosprghum*. This is the strict consensus tree of 46 equally parsimonious trees of 1666 steps (CI = 0.873) for the combined Adh1, ITS1 and ndhF sequence data under maximum parsimonious analyses. Numbers above branches are percentages of 10,000 bootstrap replicates in which the clade was recovered. Trees were rooted using *Zea mays* and *Hordeum vulgare.* Chromosome numbers sourced from [42] except those denoted with * which were sourced from [43].

Currently, there are 25 recognized species within the *Sorghum* genus, separated into 5 morphologically distinct sub-genera: *Eusorghum, Chaetosorghum, Heterosorghum, Parasorghum* and *Stiposorghum* (Figure 1) [33,34,35]. *Sorghum bicolor*, all *S. bicolor* subspecies and landraces, domesticated *Sorghum* varieties and interspecific naturalized domesticated × wild hybrids belong to the *Eusorghum*. The 4 other subgenera contain 19 species, all wild relatives in the tertiary gene pool of *S. bicolor*: the *Chaetosorghum* and *Heterosorghum* each contain a single native Australian species, *S. macrospermum* and *S. laxiflorum*, respectively; and the *Parasorghum* subgenus contains 7 species, 5 are native to Australia (*S. grande, S. leiocladum, S. matarankense, S. nitidum* and *S. timorense*) and 2 are native to Africa and Asia (*S. purpureosericeum* and *S. versicolor*). The *Stiposorghum* contains ten species all endemic to northern Australia (*S. amplum, S. angustum, S. brachypodum, S. bulbosum, S. ecarinatum, S. exstans, S. interjectum, S. intrans, S. plumosum* and *S. stipoideum*) [36]. All species of *Eusorghum* are thought to produce dhurrin at similar concentrations [37,38]. The cyanogenic status of a subset of crop wild relative (CWR) of sorghum has been studied [39,40,41]; however, there are no published studies on the degree and extent of cyanogenesis across all undomesticated species in the genus *Sorghum.*

*S. bicolor* was originally domesticated in northeastern Africa, including Ethiopia, Sudan and East Africa, around 6000 years ago [44,45], and most *Eusorghum* are also native to the African continent. *S. halepense* is thought to have originated in the Mediterranean, but has become widespread across the world and is considered an invasive weedy species [46]. The hybrid *S. × almum* was developed in Argentina from a cross between *S. bicolor* and *S. halepense* and is widely grown in Argentina for animal fodder, but it is less popular elsewhere due to high HCNp [47]. A large proportion of undomesticated *Sorghum* species (15 of 19) are distributed exclusively in the remote, relatively undisturbed regions of northern Australia [36,48,49]. The only species that is found as far south as Victoria is *S. leiocladum*, which is thought to have been traded by Australia’s First Peoples. Due to the varied geographic distribution and likely extensive period of genetic isolation between species of the *Sorghum* genus [23], the endemic Australian species provide a unique opportunity to investigate the evolutionary drivers for the deployment of cyanogenic glucosides, i.e., differences in composition that may have arisen as a result of natural selection rather than anthropogenic artificial selection.

Resource allocation theories predict that the synthesis and maintenance of nitrogen-based cyanogenic glucosides must come at a cost to plant growth [50,51]. Accordingly, plants distributed in natural, resource-limited environments, such as undomesticated *Sorghum,* might show a reduced capacity for cyanogenic glucoside production and subsequent release of HCN. In this study, a phenotype–genotype approach is employed with the aim of constructing the first profile of cyanogenesis across the undomesticated species of the *Sorghum* genus. Phenotypic variation in cyanogenic status is assessed through the analysis of differences in two distinct parameters: firstly, the quantitative potential to release hydrogen cyanide from leaf tissue is measured at different developmental stages in species of the five *Sorghum* subgenera; secondly, the identity and relative content of any cyanogenic glucosides present are determined by LC-MS. Phenotypic analyses are conducted at pre-flowering stages, as dhurrin concentration peaks during seedling development in *S. bicolor* [52]. Genotypic differences are also investigated by analyzing structural variation in genes known or thought to be involved in one of several cyanogenic pathways, including dhurrin biosynthesis and bio-activation [25,53,54], HCN detoxification and endogenous recycling of dhurrin [25,55]. In addition, the sequence of *CYP79A1,* the key gene in dhurrin biosynthesis, is analyzed in greater detail by isolation of the gene from the majority of CWR.

## 2. Results

### 2.1. Hydrogen Cyanide Potential in the Wild Relatives of Sorghum

**Figure 2:**
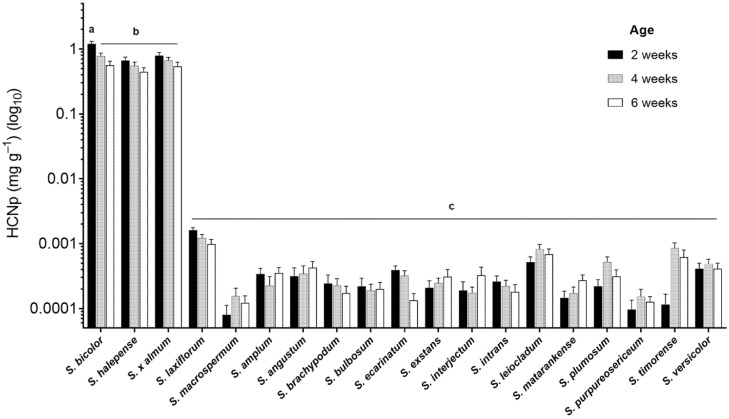
Hydrogen cyanide potential (HCNp) in dried, finely ground leaf tissue of 19 *Sorghum* species at three time points during plant development plotted on a logarithmic scale. Columns with the same letter superscript are not significantly different (*p* < 0.05).

Hydrogen cyanide potential (HCNp), as a proxy for cyanogenic glucoside concentration, was determined for leaf tissue harvested from *S. bicolor* and 18 related *Sorghum* species. Hydrogen cyanide release was measured in the leaves of all species at three time points during plant development (Figure 2). Foliar HCNp was extremely low in all wild species from the *Chaetosorghum, Heterosorghum, Parasorghum* and *Stiposorghum* subgenera compared with the three *Eusorghum* species at 2, 4, and 6 weeks post-germination (*p* < 0.001). The overall average HCNp across the tertiary species was similar at each harvest point with an average of 0.33 ± 0.04 µg g^−1^ at 2 weeks post-germination, 0.37 ± 0.03 µg g^−1^ at 4 weeks, and 0.33 ± 0.03 µg g^−1^ at 6 weeks (Figure 2). This was up to three orders of magnitude lower than the HCNp in the three *Eusorghum* species, which together had an overall average of 880 ± 70 µg g^−1^ at 2 weeks, 660 ± 50 µg g^−1^ at 4 weeks, and 520 ± 0.50 µg g^−1^ at 6 weeks (Figure 2). HCNp did not vary significantly among the *Eusorghum* species at the different time points, except for at 2 weeks when *S. bicolor* had significantly higher HCNp (1200 ± 110 µg g^−1^) than both *S. halepense* (670 ± 8 µg g^−1^) and *S.* × *almum* (790 ± 90 µg g^−1^) (*p* < 0.05). There was a general trend towards a decrease in HCNp over time in each *Eusorghum* species, though the differences were not statistically significant in either *S. halepense* or *S.* × *almum* (*p* > 0.05). While HCNp varied significantly among the 16 CWR at the µg scale, the differences were minute in terms of final HCN concentration.

### 2.2. Relative Cyanogenic Glucoside Content

**Figure 3:**
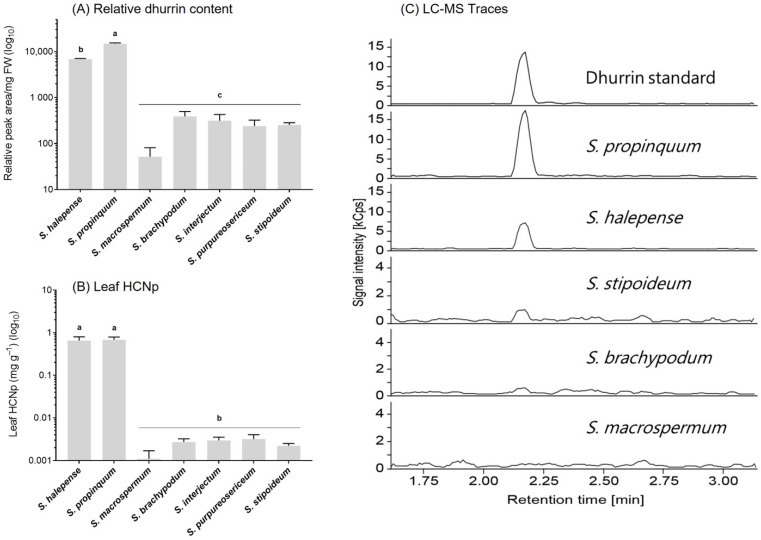
Relative dhurrin content and hydrogen cyanide potential (HCNp) in the youngest mature leaf of seven *Sorghum* species at 6 weeks post-germination plotted on a logarithmic scale. (**A**) Relative dhurrin content based on LC-MS analyses. (**B**) Leaf HCNp. (**C**) Selected LC-MS spectra from wild sorghum species depicting the differences in signal intensity of the base peak upon analysis of the same amount of fresh-frozen leaf material (RT dhurrin: 2.17 min; dhurrin parent ion at 307.1 (M-27+Na), diagnostic fragments at *m*/*z* 212.1, 185.0 and 145.0 with the *m*/*z* 185.0 fragment representing the base peak). Graphs show mean ± 1 SE (*n* = 3). Columns with different letters are statistically significant (*p* < 0.05).

LC-MS analysis of leaf tissue of a subset of five wild species (Chaetosorghum: *S. macrospermum*; Stiposorghum: *S. brachypodum*; *S. interjectum*; Parasorghum: *S. purpureosericeum*, *S. stipoideum*) and two Eusorghum species (*S. halepense* and *S. propinquum*) identified the cyanogenic glucoside present in the wild sorghum species as dhurrin, the same cyanogenic glucoside as in the cultivated sorghum *S. bicolor* (Figure 3). The two Eusorghum species showed significantly higher relative dhurrin concentrations than any of the wild species, up to two orders of magnitude in some cases (*p* < 0.05) (Figure 3A). Amongst the wild species, *S. macrospermum* had the lowest relative dhurrin concentration, although the differences were not statistically significant (Figure 3A). The pattern seen in the results from the LC-MS was similar to the HCNp detected in leaves from the same individual plants of each species (Figure 3B). Both Eusorghum species showed significantly higher HCNp, determined by the colorimetric assay compared to the wild species.

### 2.3. Variation within Cyanogenesis Related Genes in Sorghum Detected by Genome Sequencing

Access to preliminary genome sequence data for six CWR (*S. laxiflorum*, *S. macrospermum*, *S. brachypodum*, *S. leiocladum*, *S. matarankense* and *S. purpureosericeum* [35]) enabled the analysis of the variation present in selected genes involved in cyanogenesis and related pathways. Briefly, the trimmed reads were mapped to the genomic sequences of the 18 selected *S. bicolor* genes (Supplementary Table S2). The alignment of the reads identified numerous single nucleotide variants (SNVs) in all selected genes in the six species (Table 1). In *CYP79A1*, the gene encoding the enzyme catalyzing the rate-limiting step in dhurrin biosynthesis, *S. macrospermum* (*Chaetosorghum*) had the fewest SNVs (146) and *S. leiocladum* (*Parasorghum*) the most (406). This is not an unexpected result, as these are the most closely related and one of the more distantly related species, respectively, to *S. bicolor* and the *Eusorghum* according to the current phylogeny (Figure 1) [33]. In general, the sequences of the genes more directly involved in cyanogenic metabolism, including dhurrin biosynthesis (*CYP79A1, CYP71E1, UGT85B1, POR*) and bioactivation (*DHR*s, *HNL*), varied the most across all six species. Genes encoding enzymes that function in more fundamental metabolic processes, such as ethylene synthesis (*ACC*), were generally more highly conserved across all species. Interestingly, the glutathione S-transferase (*GST*) family genes thought to be involved in the endogenous recycling and detoxification of cyanogenic glucosides [55] were also relatively conserved compared to the biosynthetic and bioactivation genes. The nitrilase 4 class (*NIT4*) genes, likely involved in recycling and in the general HCN detoxification pathway, varied substantially in all species relative to *S. bicolor*.

*CYP79A1* was analyzed in greater detail by examining the predicted effects of all SNVs on the protein sequence. The majority of SNVs were predicted to result in a synonymous variant in the amino acid sequence and to have little or no impact on CYP79A1 function. The remaining SNVs were predicted to have a moderate impact on the protein as a result of missense mutations, although the majority of amino acid changes occurred within the hydrophobic class and are likely to have a lower impact on protein function [56]. Overall, 45% of this subset of SNVs resulted in changes among positively charged amino acids.

### 2.4. Variation within the Key Biosynthesis Gene, CYP79A1

To investigate the sequence of *CYP79A1* in greater detail, PCR was used to amplify the gene from the majority of the wild sorghum species (Table 2). Primers were designed to the coding region of *CYP79A1* as it was expected that untranslated regions would be more variable. PCR was successful in amplifying the full length *CYP79A1* gene from 11 species; for 3 species, data were available for both genome and PCR approaches (Table 2). Alignment of the CYP79A1 amino acid sequences obtained indicates that, overall, there is a high degree of conservation across the sorghum genus (approx. 85–95% identity; Supplementary Figure S1). The PCR sequence data show that the coding region of the CYP79A1 sequence in the wild sorghum species varies between 550 and 559 amino acid residues compared to 559 amino acids in *S. bicolor*. The length of the single intron in *CYP79A1* varied from 81 to 195 nucleotide residues (Table 2). Significant divergence in the sequence of the introns and other non-coding regions may be less easily detected when mapping Illumina reads to the *S. bicolor* reference as highly divergent reads may not map.

**Figure 4:**
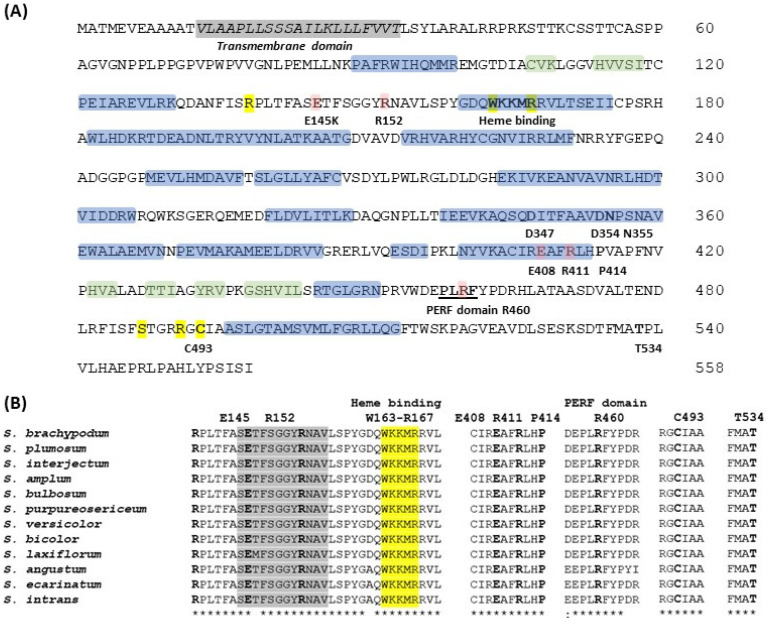
(**A**) Complete amino acid sequence of CYP79A1 from *S. bicolor* with α-helixes and β-sheets. Conserved regions and specific amino acids important for CYP79A1 function are marked. Blue—α-helixes; green—β-sheets; yellow—key amino acids, pink—site directed mutagenesis [12,57,58,59,60,61,62]. (**B**) Selected regions of the CYP79A1 sequence of wild *Sorghum* species showing high conservation, denoted by *. Alignment of complete sequences is shown in Supplementary Figure S1.

Homology models of CYP79A1 were made using the solved crystal structures of relevant P450s as a template [57,58]. CYP79A1 contains 12 conserved major α-helices and 6 β-strands forming 2 highly conserved β-sheets (Figure 4A). The correct folding of the CYP79A1 protein is important for functionality, ensuring heme-binding and correct docking of the substrate, tyrosine. Modelling and mutation studies also identified additional key amino acids for CYP79A1 activity; the R152 residue (*S. bicolor* sequence used for numbering) is involved in positioning the tyrosine substrate [57,58] whilst R411 is part of the E-R-R triad locking the heme pockets into position and stabilizing the protein core structure by formation of a salt bridge with E408 and R411 of the XEXXR sequence and R460 in the PERF motif (a P450 signature sequence) [59]. The heme-binding domain (WXXXR) is also important for correct folding and stability of the enzyme. Site directed mutagenesis and the generation of sorghum mutants also identified specific amino acids that impact on enzyme function and the synthesis of dhurrin [58,60]. The mutations E145K, R152A and T534A resulted in reduced dhurrin synthesis [58], whilst sorghum lines with mutations at P414L and C493Y resulted in acyanogenic plants [60,61]. The analysis of the sequence data obtained by PCR from the wild species indicated that these identified motifs and key amino acids are conserved in all the wild species investigated (Figure 4B). The high conservation of the coding sequences in the *CYP79A1* gene may reflect that this gene has an essential conserved function and supports the results of the cyanide assays, indicating that dhurrin is synthesized to some degree in all species.

## 3. Discussion

The current study investigates cyanogenesis in most of the currently known tertiary wild relatives of *S. bicolor* for the first time. The cyanogenic glucoside present in the wild species and elite *S. bicolor* is dhurrin, and the genomic machinery encoding the biosynthetic enzymes catalyzing dhurrin production and bioactivation was highly conserved among all tested species across the *Sorghum* genus. CYP79A1 has a very high substrate specificity with tyrosine being the only amino acid used as substrate [63]. With the highly conserved CYP79A1 sequences found in the wild compared to elite sorghums, it is not surprising that the wild sorghums also produce dhurrin. However, the phenotypic expression of hydrogen cyanide potential (HCNp) was substantially reduced in all species from the four undomesticated subgenera compared to *S. bicolor* and the domesticated *Eusorghum*. While a lower cyanogenic capacity in the tertiary *Sorghum* species was not unexpected, the order of magnitude differences between cultivated and tertiary *Sorghum* was greater than anticipated. Moreover, because of the high sequence identity of *CYP79A1* between the elite and wild sorghum lines, this is not likely to result from the lost catalytic capacity of the CYP79A1 enzyme in the wild species.

### 3.1. Phenotypic Variation of Cyanogenesis in Sorghum

In the current study, *S. bicolor* and the other tested *Eusorghum* species (*S.* × *almum, S. halepense* and *S. propinquum*) showed high HCNp and dhurrin content. The production of specialized metabolites, such as cyanogenic glucosides, has long been thought to come at a metabolic cost to plants, tying up resources that could otherwise be utilized in growth and development [50,51]. Under the relatively controlled environmental conditions characterizing cultivated systems, these costs may be partially offset by a more stable uptake of essential resources, such as water and soil nutrients. However, production costs are likely to be more keenly felt in highly variable natural environments. This may be reflected in the highly reduced dhurrin content and negligible HCNp detected in the undomesticated species of the *Chaetosorghum, Heterosorghum, Parasorghum* and *Stiposorghum* subgenera. A large proportion of species in these groups are endemic to northern Australia, with the main center of diversity extending from the northerly monsoonal tropics to the arid and semi-arid regions of Central Australia. Soils in these regions are typically characterized by low concentrations of available nitrogen [23], the signature element and part of the fundamental nitrile group present in cyanogenic glucosides. This could suggest that the endemic wild *Sorghum* species with their low HCNp prioritize the allocation of available nitrogen to general metabolic processes in growth and development rather than to the production of dhurrin. In future, a comparative study of the cyanogenic glucoside content in the elite and wild *Sorghum* species from different habitats and following subjection to different levels of nitrogen deficiency might provide insights into the importance of these parameters in individual species belonging to the same genus [39,40].

Leaf tissue of wild species expressed cyanide potential at a scale lower than 1 µg g^−1^, translating to dhurrin concentrations of less than 1 ppm. For feeding herbivores, plant tissue containing dhurrin at this low scale would be virtually indistinguishable from tissue with no capacity to release HCN, i.e., functionally acyanogenic [64]. Unless cyanogenesis is specifically induced by insect or pest attacks, this suggests that the wild, undomesticated *Sorghum* species do not utilize cyanogenic glucosides as part of their chemical defense, particularly as leaves are considered the plant organ most vulnerable to predation. Production of dhurrin in *S. bicolor* is developmentally regulated, with concentrations often found to be highest in young, developing tissues, such as newly formed leaves [52,65,66,67,68,69]. In terms of cyanogenesis, this pattern is consistent with the optimal resource allocation theory, in that leaves are generally the most heavily defended organ as they house the photosynthetic apparatus [70,71]. With the potentially high costs of dhurrin synthesis in nitrogen-poor environments, the wild *Sorghum* species may instead place greater emphasis on other defense mechanisms to deter herbivores, including the production of less costly carbon-based physical structures, such as trichomes [72,73,74]. Indeed, a higher density of epidermal trichomes has been documented *S. brachypodum* and *S. macrospermum* [40]. It is currently unclear which specific herbivores feed on the endemic Australian species, although marsupials have been seen feeding on some species in the field (pers. comm. Dr Sally Norton). Recent evidence indicates that cyanogenic glucosides possess additional physiological functions beyond defense, in particular acting as storage compounds for reduced nitrogen that can be recovered for use in general plant metabolism upon demand [25,55,67,75] In the wild *Sorghum* species, dhurrin may be turned over immediately after production to release freely available reduced nitrogen. Future studies could explore this by comparative studies of the transcript levels of the genes encoding the recycling pathway and measuring the biosynthetic activity of the biosynthetic enzymes in microsomes isolated from the wild and domesticated sorghum lines, also considering the importance of diurnal rhythms [55,75,76].

An extremely low concentration of dhurrin and the resulting minute HCN potential were consistently observed across all wild *Sorghum* species examined in the current study. In previous studies, high quantitative and qualitative intraspecific variation of cyanogenesis has been recorded within species of other plant genera [3]. Some species of *Eucalyptus* show high quantitative variation for cyanogenic traits both within and between different populations [64,77,78,79]. In several species of *Lotus,* the potential for HCN release has been observed to vary both quantitatively between individual plants, and qualitatively at the population scale [29,80,81,82]. Within natural populations of white clover (*Trifolium repens*), individual plants can be either cyanogenic or completely acyanogenic, representing the true polymorphism of the trait [28,80,83,84,85]. In cassava (*Manihot esculenta*), another major cyanogenic crop, genetically isolated wild relatives (equivalent to the tertiary *Sorghum* species) showed quantitative variation in their potential for HCN release in tubers under stable environmental conditions [26,27]. Such levels of intraspecific variation were not apparent in the wild *Sorghum* species. However, different populations may vary substantially in the capacity to produce dhurrin. In our current study, only a single accession of each species, i.e., a wild population from a single locality, was examined.

### 3.2. Genomic Variation of Cyanogenesis in Sorghum

The preliminary genome sequencing analysis reported in this study focused on the genetic variation in cyanogenic metabolism within six wild relatives of *Sorghum*. The structure of the CYP79A1 gene was further investigated by isolating and sequencing the gene from the majority of sorghum CWRs. These results and the single nucleotide variants (SNVs) analysis of the genome data suggest that the majority of amino acid changes identified in the wild species do not have a major effect on the catalytic activity of CYP79A1, the important initial step of dhurrin biosynthesis.

In the analysis of selected cyanogenic genes in wild *Sorghum*, differences in gene copy number and ploidy levels may have major effects. For example, a *CYP79A1*-like gene positioned at chromosome 10 shows 75% identity to *CYP79A1* positioned at chromosome 1 in *S. bicolor* [86]. When full genome sequences of the wild *Sorghum* species become available, more detailed analysis of the *CYP79* sequences present should be performed to examine whether the sequence reads have been assigned to the correct gene. The ploidy level of the different *Sorghum* species also varies with the chromosome number for *S. bicolor* 2n = 20, whilst for the wild species it varies with 2n = 10–40, potentially affecting the synthesis and/or recycling of dhurrin.

The PCR and genomic sequencing results analyzed to date show that the dhurrin biosynthetic genes are present and largely intact in the geographically and genetically isolated tertiary *Sorghum* species. However, there were substantial differences in HCNp and concentration of dhurrin between wild and domesticated *Sorghum*. Therefore, the expression of the key dhurrin biosynthesis gene *CYP79A1* is likely to be controlled by regulatory mechanisms. It has previously been shown that the biosynthesis of dhurrin in *S. bicolor* is regulated at the transcriptional level [52]. Ehlert et al. [12] also found evidence of transcriptional regulation of the cyanogenic glucoside epiheterodendrin in barley. Wild almond species accumulate the phenylalanine-derived bitter and toxic cyanogenic glucoside amygdalin [87]. Almond domestication resulting in sweet kernels resulted from a single amino acid substitution (L346P) in the transcription factor bHLH2 controlling the transcription of the CYP79- and CYP71-encoding genes in the amygdalin pathway. This nonsynonymous point mutation in the dimerization domain of bHLH2 prevented the formation of a functional dimer and the transcription of the two biosynthetic genes [13]. The absence of dhurrin formation in the leaves of the wild sorghum species is thus likely also to reflect the lack of transcription of the *CYP79A1* gene. Future studies should include transcriptomic analysis of the tertiary wild *Sorghum* species at different stages of plant development, in different tissue types, and in plants grown under different environmental conditions to further understand the regulation of dhurrin. The genomic and phenotypic variation apparent in this one functional trait, cyanogenesis, suggests a high degree of genetic diversity in the wild *Sorghum* germplasm. These species therefore shape as a valuable genetic resource for the breeding of more climate-resilient *Sorghum* crops in the future [23,88].

## 4. Materials and Methods

### 4.1. Plant Material and Growing Conditions

Seeds from accessions of 15 undomesticated/wild Sorghum species from subgenera Chaetosorghum, Heterosorghum, Parasorghum and Stiposorghum (Chaetosorghum: *S. macrospermum* E.D. Garber; Heterosorghum: *S. laxiflorum* F.M. Bailey; Parasorghum: *S. leiocladum* (Hack.) C.E. Hubb., *S. purpureosericeum* (Hochst. Ex A. Rich.) Asch. & Schweinf. and *S. versicolor* Andersson.); Stiposorghum: *S. amplum* Lazarides, *S. angustum* S.T. Blake, *S. brachypodum* Lazarides, *S. bulbosum* Lazarides, *S. ecarinatum* Lazarides, *S. exstans* Lazarides, *S. interjectum* Lazarides, *S. intrans* F. Muell. Ex Benth., *S. plumosum* (R.Br) P. Beauv., *S. stipoideum* (Ewart & Jean White) C.A. Gardner & C.E. Hubb), and three Eusorghum species (*S. propinquum* (Kunth) Hitchc, *S. halepense* (L.) Pers. and *S.* × *almum* Parodi), were obtained from the Australian Grains Genebank (AGG), Horsham, Victoria (Supplementary Table S1). *Sorghum bicolor* (BTx623) seeds were supplied by the Queensland Alliance for Agriculture and Food Innovation (QAAFI), University of Queensland (UQ).

Seeds of the wild sorghums used for all experiments were germinated as detailed in Cowan et al. 2020 [41]. Briefly the caryopsis was removed from the seed covering and placed in 3 mM gibberellic acid (GA_3_) (Growth Regulator, G7645, Sigma-Aldrich, St. Louis, MO, USA) and left at room temperature (24 °C) for approximately 15 h, transferred to sterile moist filter paper and incubated at 35 °C during the day (9 a.m.–5 p.m.) and 25 °C overnight, with a 14 h photoperiod. Seedlings were transplanted into a soil and perlite mix (4:1 ratio). All plants analyzed for dhurrin were grown under controlled greenhouse conditions at Monash University (coordinates: 37°54′36″ S, 145°08′02″ E) with a mean temperature of 27.8 °C ± 2.6 °C and 18.3 °C ± 2.1 °C day/night, respectively, and an average photoperiod of 14 h (average photosynthetic photon flux density: 421 ± 71 µmol quanta m^–2^ s^–1^). Supplementary light from sodium lamps (MK-1 Just-a-shade, Ablite Australia) was used to maintain the irradiation level when the natural photoperiod decreased over autumn. The plants used for DNA extraction for PCR amplification and sequencing of *CYP79A1* were grown at both Monash University and the Australian Grains Genebank, Horsham, VIC, Australia (36°43′21.93764″ S and 142°10′29.50331″ E) under the conditions detailed above.

### 4.2. Hydrogen Cyanide Assays

The youngest fully emerged leaf was harvested from each plant at 2, 4 and 6 weeks for analysis of cyanogenic glucoside concentration. The leaf tissue was freeze dried, ground to a fine powder using a MM 300 MixerMill (Retsch, Haan, Germany) and 10 mg of tissue was used to determine the evolved HCN detection method following Gleadow et al. [89]. The hydrogen cyanide potential (HCNp) is the total amount of HCN evolved from hydrolysis of the entire content of endogenous cyanogenic glucosides. It is used as a proxy for dhurrin, such that each mg of HCN is equivalent to 11.5 mg of dhurrin in the plant tissue. All assays were performed in triplicate, and NaCN standards were included on each plate to create a standard curve. Data are expressed as cyanide potential (HCNp, mg CN g^−1^ dry weight), that is, the maximum cyanide release per mg cyanogenic glucoside and includes any free cyanide that may be present in the tissue. Depending on the amount of HCNp detected, the samples were either diluted 1 in 10 or assayed without dilution.

### 4.3. LC-MS Analysis and Identification of Cyanogenic Glucoside(s)

The youngest fully emerged leaf from plants of individual accessions of Sorghum species (Chaetosorghum: *S. macrospermum*; Stiposorghum: *S. brachypodum*, *S. interjectum*; Parasorghum: *S. purpureosericeum*, *S. stipoideum*; Eusorghum: *S. halepense*, *S. propinquum*) (*n* = 3) was removed at the ligule at six weeks post-germination, snap frozen and shipped on dry ice to the University of Copenhagen for analysis. The presence and identification of cyanogenic glucosides were analyzed using tandem mass spectrometry similar to Montini et al. 2020 [90]. Briefly, chromatography was performed on an Advance UHPLC system (Bruker, Bremen, Germany). Separation was achieved on a Zorbax XDB-C18 column (3.0 × 100 mm, 1.8 µm, Agilent Technologies). Formic acid (0.05%, *v*/*v*) in water and acetonitrile (supplied with 0.05% formic acid, *v*/*v*) were employed as mobile phases A and B, respectively. Mobile phase flow rate was 500 µL min^−1^, and the elution profile was as follows: 0–0.3 min, 2% B; 0.3–0.9 min, 2–15% B; 0.9–1.4 min, 15–60% B; 1.4–3.3 min 60–100% B; 3.3–3.9 min, 100% B; 3.9–4.0 min 100–2% B and 4.0–5.0 min 2% B. The column temperature was maintained at 40 °C. The liquid chromatograph was coupled to an EVOQ Elite TripleQuadrupole mass spectrometer (Bruker, Bremen, Germany) equipped with an electrospray ion source (ESI) operated in positive ion mode. The ion spray voltage was maintained at 5000 V. The cone temperature was set to 300 °C and cone gas to 20 psi. The heated probe temperature was set to 200 °C and probe gas flow set to 50 psi. The nebulizing gas was set to 60 psi and collision gas to 1.6 mTorr. Multiple reaction monitoring (MRM) was used to monitor analyte precursor ion → fragment ion transitions. MRM transitions and corresponding collision energies (CE) were determined from direct infusion experiments of a reference standard. Dhurrin was detected as the sodium adduct [M+Na]^+^ with the following MRM transitions: *m*/*z* 334.1 → 145.0 (CE −15 eV), *m*/*z* 334.1 → 185.0 (CE −15 eV), *m*/*z* 334.1 → 307.0 (CE −10 eV); Both, Q1 and Q3 quadrupoles were maintained at unit resolution. The Bruker MS Workstation software (Version 8.2,1) was used for data acquisition and processing. The relative quantities of dhurrin were calculated as the ratio of the base peak area (*m*/*z* 185) to the sample weight (50 mg). Chemically synthesized dhurrin was used as a standard [91].

### 4.4. Variant Analysis within Selected Genes Involved in Cyanogenesis and Related Pathways

The available sequences of a series of selected genes in the genomes of 6 wild sorghum species [35] were used to analyze the variation in 18 genes selected based on known and putative roles in cyanogenesis (dhurrin biosynthesis, bioactivation, recycling) as well as in the synthesis of tyrosine (the amino acid substrate for the first step in dhurrin biosynthesis) and ethylene (Supplementary Table S2). The *S. bicolor* genomic sequences for these genes were downloaded to CLC from Phytozome (https://phytozome.jgi.doe.gov, accessed on 10 January 2022) and used to map the reads generated from the partial sequencing of the six wild Sorghum species. Single nucleotide variants (SNVs) for each gene were called using the basic variant detection tool in CLC Genomics Workbench 12.0 (CLC Bio, Aarhus, Denmark) with a minimum coverage of 10, read count of 2 and allele frequency of 10%. Multi-allelic nucleotide variations were not included in this study as these were likely to be false positives, potentially caused by sequencing errors or errors in variant detection. Predicted effects on the protein sequence of all SNVs for all species were investigated in *CYP79A1*, the gene encoding the rate-limiting step of dhurrin biosynthesis [52], using the online Ensembl Variant Effect Predictor software [92]. The impact of these changes on the amino acid sequence of CYP79A1 and protein function was determined using the online software program SNAP2 within PredictProtein (https://www.predictprotein.org/, accessed on 10 January 2022) [56].

### 4.5. Detailed Sequence Analysis of CYP79A1 across the Sorghum Phylogeny

To further analyze the *CYP79A1* sequence variation, polymerase chain reaction (PCR) was used to amplify the gene from additional wild sorghum species. DNA was extracted from leaf tissue using the CTAB protocol [93] and the concentration and integrity determined by gel electrophoresis and NanoDrop spectrophotometer (ND-1000, Thermo Fisher Scientific). The sequence of *S. bicolor* was used to design primers to amplify the full coding region of *CYP79A1* and synthesized by Sigma (https://www.sigmaaldrich.com/AU/en, accessed on 10 January 2022) (Supplementary Table S3). High fidelity *Pfu* DNA polymerase (Promega) was used in the PCR (according to manufacturer’s instructions) with touchdown cycling conditions as follows: 95 °C—4 min; 95 °C—30 s step down 62 °C, −0.5 °C/cycle for 9 cycles—30 s, 72 °C—2.5 min; 95 °C—30 s, 58 °C—30 s, 72 °C—2.5 min for 29 cycles; 72 °C—10 min; 4 °C hold. PCR products were purified using the Wizard gel purification kit (Promega) and sequenced by Micromon (Monash University). Additional internal primers were used for complete sequencing of the 2 kb region (Supplementary Table S3). Sequences were analyzed using Snapgene, Clustal Omega, NCBI blast and Phytozome.

### 4.6. Statistical Analysis

All quantitative data were analyzed using GraphPad Prism version 7.02 for Windows (GraphPad Software, San Diego, CA, USA). Ordinary one-way analysis of variance (ANOVA), followed by Tukey’s multiple comparisons tests were used to compare HCNp between and within species across different time points. A 95% confidence level was set for all statistical tests.

## 5. Conclusions

Cyanogenesis is a highly variable functional trait, controlled by a complex interaction of internal and external factors [3]. The current study took the fundamental initial step of characterizing cyanogenesis in the undomesticated species of the genus Sorghum. While the structural genetic variation of the cyanogenic machinery was limited, all tested species of *Chaetosorghum*, *Heterosorghum*, *Parasorghum* and *Stiposorghum* showed negligible potential for HCN release in leaf tissues. In simple terms, this low phenotypic expression of cyanogenesis might reflect the conditions in their natural environments, such as a limited access to nutrient resources and/or differences in herbivore pressures [79]. The reality is likely to be much more complex. The regulation of dhurrin and HCNp is also dependent on plant ontogeny and specific tissue type in *S. bicolor* [65,67,94]. In order to better understand the potential utilization and growth-defense trade-offs of cyanogenic glucosides in general plant metabolism, detailed tissue- and age-dependent variation of HCNp in wild species in comparison to *S. bicolor* is required.

## Figures and Tables

**Table 1:** Number of single nucleotide variants (SNVs) called for the examined genes in the six wild *Sorghum* species—*S. laxiflorum* (lax), *S. macrospermum* (mac), *S. brachypodum* (bra), *S. leiocladum* (lei), *S. matarankense* (mat) and *S. purpureosericeum* (pur)—when mapped to the genomic *S. bicolor* sequence of the selected genes.

	*Sorghum* Species					
Gene	Lax	Mac	Bra	Lei	Mat	Pur
*CYP79A1*	285	146	258	406	338	235
*CYP71E1*	130	306	270	232	232	157
*UGT85B1*	157	104	99	155	177	154
*POR*	295	331	322	297	233	234
*NIT4A*	167	237	248	198	181	149
*NIT4B1*	321	403	493	187	344	356
*NIT4B2*	186	330	314	224	174	191
*GST1*	116	54	150	42	106	109
*GST1B*	134	138	162	132	138	116
*GST3*	41	81	69	61	52	58
*CAS C1*	148	98	247	119	123	132
*CAS26*	125	250	205	156	139	147
*MATE*	222	188	165	188	177	155
*HNL*	342	251	528	437	297	386
*DHR1*	216	329	477	130	206	354
*DHR2*	183	332	429	86	100	110
*ACC*	27	63	70	34	33	75
*CM7*	109	265	231	119	119	136

**Table 2:** Sequence details of *CYP79A1* from sorghum tertiary crop wild relatives. Either PCR or genome sequencing data were analyzed.

Species	Source of Sequence Data	Coding Sequence (bp)	Peptide Sequence (aa)	Intron Length (bp)	
*S. bicolor*	database	1677	559	97	Phytozome
*S. amplum*	PCR	1665	555	87	
*S. angustum*	PCR	1653	551	94	
*S. brachypodum*	genome	1677			
*S. brachypodum*	PCR	1662	554	99	
*S. bulbosum*	PCR	1671	557	92	
*S. ecarinatum*	PCR	1656	552	82	
*S. exstans*	PCR	977	338	-	Partial only
*S. interjectum*	PCR	1665	555	81	
*S. intrans*	PCR	1665	555	105	
*S. laxiflorum*	genome	1677			
*S. laxiflorum*	PCR	1662	554	123	
*S. leiocladum*	genome	1677			
*S. macrospermum*	genome	1677			
*S. matarankense*	genome	1677			
*S. plumosum*	PCR	1668	556	195	
*S. propinquum*	-				No tissue
*S. purpureosericeum*	genome	1677			
*S. purpureosericeum*	PCR	1650	550	163	
*S. stipoideum*	-				No amplification
*S. timorense*	-				No amplification
*S. versicolor*	PCR	1662	554	177	

## Data Availability

Data is available from the authors on request.

## Supplementary Materials

The following are available online at https://www.mdpi.com/article/10.3390/genes13010140/s1. Figure S1: CLUSTAL O (1.2.4) multiple sequence alignment of the amino acid sequences of CYP79A1 obtained by PCR from wild sorghum species; Table S1: Accession and provenance details of the sorghum crop wild relatives; Table S2: Genes selected for variant analysis; Table S3: Sequence of the primers used to amplify *CYP79A1* from the wild sorghum species.

## References

[1] Sage R.F., Zhu X.-G. (2011). Exploiting the engine of C_4_ photosynthesis. J. Exp. Bot..

[2] Tari I., Laskay G., Takács Z., Poór P. (2013). Response of sorghum to abiotic stresses: A review. J. Agron. Crop Sci..

[3] Gleadow R.M., Møller B.L. (2014). Cyanogenic glycosides: Synthesis, physiology, and phenotypic plasticity. Annu. Rev. Plant Biol..

[4] Morant A.V., Jørgensen K., Jørgensen C., Paquette S.M., Sánchez-Pérez R., Møller B.L., Bak S. (2008). β-Glucosidases as detonators of plant chemical defense. Phytochemistry.

[5] Heraud P., Cowan M.F., Marzec K.M., Møller B.L., Blomstedt C.K., Gleadow R. (2018). Label-free Raman hyperspectral imaging analysis localizes the cyanogenic glucoside dhurrin to the cytoplasm in sorghum cells. Sci. Rep..

[6] Zagrobelny M., Bak S., Møller B.L. (2008). Cyanogenesis in plants and arthropods. Phytochemistry.

[7] Gleadow R.M., Woodrow I.E. (2002). Constraints on effectiveness of cyanogenic glycosides in herbivore defense. J. Chem. Ecol..

[8] Tattersall D.B., Bak S., Jones P.R., Olsen C.E., Nielsen J.K., Hansen M.L., Høj P.B., Møller B.L. (2001). Resistance to an herbivore through engineered cyanogenic glucoside synthesis. Science.

[9] Ballhorn D.J., Kautz S., Heil M., Hegeman A.D. (2009). Cyanogenesis of wild lima bean (*Phaseolus lunatus* L.) Is an efficient direct defence in nature. PLoS ONE.

[10] Wheeler J.L., Mulcahy C., Walcott J.J., Rapp G.G. (1990). Factors affecting the hydrogen cyanide potential of forage sorghum. Aust. J. Agric. Res..

[11] Andersen M.D., Busk P.K., Svendsen I., Møller B.L. (2000). Cytochromes P-450 from cassava (*Manihot esculenta* Crantz) catalyzing the first steps in the biosynthesis of the cyanogenic glucosides linamarin and lotaustralin: Cloning, functional expression in pichia pastoris, and substrate specificity of the isolated recombinant enzymes. J. Biol. Chem..

[12] Ehlert M., Jagd L.M., Braumann I., Dockter C., Crocoll C., Motawia M.S., Møller B.L., Lyngkjær M.F. (2019). Deletion of biosynthetic genes, specific SNP patterns and differences in transcript accumulation cause variation in hydroxynitrile glucoside content in barley cultivars. Sci. Rep..

[13] Sánchez-Pérez R., Pavan S., Mazzeo R., Moldovan C., Cigliano R.A., Del Cueto J., Ricciardi F., Lotti C., Ricciardi L., Dicenta F. (2019). Mutation of a bHLH transcription factor allowed almond domestication. Science.

[14] Hansen C.C., Sørensen M., Veiga T.A.M., Zibrandtsen J.F.S., Heskes A.M., Olsen C.E., Boughton B.A., Møller B.L., Neilson E.H.J. (2018). Reconfigured cyanogenic glucoside biosynthesis in *Eucalyptus cladocalyx* involves a cytochrome P450, CYP706C55. Plant Physiol..

[15] Thodberg S., Sørensen M., Bellucci M., Crocoll C., Bendtsen A.K., Nelson D.R., Motawia M.S., Møller B.L., Neilson E.H.J. (2020). A flavin-dependent monooxygenase catalyzes the initial step in cyanogenic glycoside synthesis in ferns. Commun. Biol..

[16] Gleadow R., Pegg A., Blomstedt C.K. (2016). Resilience of cassava (*Manihot esculenta* Crantz) to salinity: Implications for food security in low-lying regions. J. Exp. Bot..

[17] Bredeson J.V., Lyons J.B., Prochnik S.E., Wu G.A., Ha C.M., Edsinger E., Grimwood J., Schmutz J., Rabbi I.Y., Egesi C. (2016). Sequencing wild and cultivated cassava and related species reveals extensive interspecific hybridization and genetic diversity. Nat. Biotechnol..

[18] Jones D.A. (1998). Why are so many food plants cyanogenic?. Phytochemistry.

[19] McKey D., Cavagnaro T.R., Cliff J., Gleadow R. (2010). Chemical ecology in coupled human and natural systems: People, manioc, multitrophic interactions and global change. Chemoecology.

[20] Franzmann B.A., Hardy A.T., Murray D.A.H., Henzell R.G. (2008). Host-plant resistance and biopesticides: Ingredients for successful integrated pest management (IPM) in Australian sorghum production. Aust. J. Exp. Agric..

[21] Sharma H.C., Franzmann B.A., Henzell R.G. (2002). Mechanisms and diversity of resistance to sorghum midge, *Stenodiplosis sorghicola* in *Sorghum bicolor*. Euphytica.

[22] Fuller D.Q., Stevens C.J., Mercuri A.M., D’Andrea A.C., Fornaciari R., Höhn A. (2018). Sorghum Domestication and Diversification: A Current Archaeobotanical Perspective. Plants and People in the African Past: Progress in African Archaeobotany.

[23] Dillon S.L., Shapter F.M., Henry R.J., Cordeiro G., Izquierdo L., Lee L.S. (2007). Domestication to crop improvement: Genetic resources for *Sorghum* and *Saccharum* (Andropogoneae). Ann. Bot..

[24] Winchell F., Brass M., Manzo A., Beldados A., Perna V., Murphy C., Stevens C., Fuller D.Q. (2018). On the origins and dissemination of domesticated sorghum and pearl millet across Africa and into India: A view from the Butana group of the far eastern Sahel. Afr. Archaeol. Rev..

[25] Nielsen L.J., Stuart P., Pičmanová M., Rasmussen S., Olsen C.E., Harholt J., Møller B.L., Bjarnholt N. (2016). Dhurrin metabolism in the developing grain of *Sorghum bicolor* (L.) Moench investigated by metabolite profiling and novel clustering analyses of time-resolved transcriptomic data. BMC Genom..

[26] Nassar N.M.A., Fichtner S.S. (1978). Hydrocyanic acid content in some wild *Manihot* (cassava) species. Can. J. Plant Sci..

[27] Nassar N.M.A. (2000). Wild cassava, *Manihot* spp.: Biology and potentialities for genetic improvement. Genet. Mol. Biol..

[28] Blaim H., Nowacki E. (1979). Cyanogensis in *Lotus* and *Trifolium* species. Acta Agrobot..

[29] Blaise S., Carter D., Reynaud J. (1991). Evolution and differentiation of *Lotus corniculatus*/*Lotus alpinus* populations from French South-Western Alps. I. Morphologic and cyanogenic variations. Evol. Trends Plants.

[30] Band L., Heyn C.C., Plitmann U. (1981). Distribution of cyanogenesis in *Lotus* (Leguminosae). Taxon.

[31] Dahler J.M., McConchie C., Turnbull C.G.N. (1995). Quantification of cyanogenic glycosides in seedlings of three *Macadamia* (Proteaceae) species. Aust. J. Bot..

[32] Selmar D., Lieberei R., Junqueira N., Biehl B. (1991). Changes in cyanogenic glucoside content in seeds and seedlings of *Hevea* species. Phytochemistry.

[33] Dillon S.L., Lawrence P.K., Henry R.J., Price H.J. (2007). *Sorghum* resolved as a distinct genus based on combined ITS1, ndhF and Adh1 analyses. Plant Syst. Evol..

[34] Ananda G.K.S., Myrans H., Norton S.L., Gleadow R., Furtado A., Henry R.J. (2020). Wild sorghum as a promising resource for crop improvement. Front. Plant Sci..

[35] Ananda G., Norton S., Blomstedt C., Furtado A., Møller B.L., Gleadow R., Henry R. (2021). Phylogenetic relationships in the *Sorghum genus* based on sequencing of the chloroplast and nuclear genes. Plant Genome.

[36] Lazarides M., Hacker J.B., Andrew M.H. (1991). Taxonomy, cytology and ecology of indigenous Australian sorghums (*Sorghum* Moench: Andropogoneae: Poaceae). Aust. Syst. Bot..

[37] Nicollier G.F., Pope D.F., Thompson A.C. (1983). Biological activity of dhurrin and other compounds from Johnson grass (*Sorghum halepense*). J. Agric. Food Chem..

[38] Gray E., Rice J.S., Wattenbarger D., Benson J.A., Hester A.J., Loyd R.C., Greene B.M. (1968). Hydrocyanic Acid Potential of Sorghum Plants Grown in Tennessee.

[39] Myrans H., Vandegeer R.K., Henry R.J., Gleadow R.M. (2021). Nitrogen availability and allocation in sorghum and its wild relatives: Divergent roles for cyanogenic glucosides. J. Plant Physiol..

[40] Cowan M.F., Blomstedt C.K., Møller B.L., Henry R.J., Gleadow R.M. (2021). Variation in production of cyanogenic glucosides during early plant development: A comparison of wild and domesticated sorghum. Phytochemistry.

[41] Cowan M.F., Blomstedt C.K., Norton S.L., Henry R.J., Møller B.L., Gleadow R. (2020). Crop wild relatives as a genetic resource for generating low-cyanide, drought-tolerant Sorghum. Environ. Exp. Bot..

[42] Price H.J., Dillon S.L., Hodnett G., Rooney W.L., Ross L., Johnston J.S. (2005). Genome evolution in the genus *Sorghum* (Poaceae). Ann. Bot..

[43] Liu Q., Liu H., Wen J. (2017). Relationships between *Sorghum bicolor* (Poaceae) and its close relatives based on genomic in situ hybridization evidence. Turk. J. Bot..

[44] Winchell F., Stevens C.J., Murphy C., Champion L., Fuller D. (2017). Evidence for sorghum domestication in fourth millennium BC Eastern Sudan: Spikelet morphology from ceramic impressions of the butana group. Curr. Anthropol..

[45] De Wet J.M.J., Harlan J.R. (1971). The origin and domestication of *Sorghum bicolor*. Econ. Bot..

[46] Venkateswaran K., Elangovan M., Sivaraj N., Aruna C., Visarada K.B.R.S., Bhat B.V., Tonapi V.A. (2019). Origin, domestication and diffusion of *Sorghum bicolor*. Breeding Sorghum for Diverse End Uses.

[47] Anon *Sorghum* × *almum* Parodi: Hybrid between *Sorghum halepense* × *S. bicolor*, Poaceae. https://www.hort.purdue.edu/newcrop/duke_energy/Sorghum_Xalmum.html.

[48] Norton S.L., Khoury C.K., Sosa C.C., Castañeda-Álvarez N.P., Achicanoy H.A., Sotelo S. (2017). Priorities for enhancing the ex situ conservation and use of Australian crop wild relatives. Aust. J. Bot..

[49] Myrans H., Diaz M.V., Khoury C.K., Carver D., Henry R.J., Gleadow R. (2020). Modelled distributions and conservation priorities of wild sorghums (*Sorghum* Moench). Divers. Distrib..

[50] Herms D.A., Mattson W.J. (1992). The dilemma of plants—To grow or defend. Q. Rev. Biol..

[51] McKey D. (1974). Adaptive patterns in alkaloid physiology. Am. Nat..

[52] Busk P.K., Møller B.L. (2002). Dhurrin synthesis in sorghum is regulated at the transcriptional level and induced by nitrogen fertilization in older plants. Plant Physiol..

[53] Darbani B., Motawia M.S., Olsen C.E., Nour-Eldin H.H., Møller B.L., Rook F. (2016). The biosynthetic gene cluster for the cyanogenic glucoside dhurrin in *Sorghum bicolor* contains its co-expressed vacuolar MATE transporter. Sci. Rep..

[54] Hayes C.M., Burow G.B., Brown P.J., Thurber C., Xin Z., Burke J.J. (2015). Natural variation in synthesis and catabolism genes influences dhurrin content in Sorghum. Plant Genome.

[55] Bjarnholt N., Neilson E.H.J., Crocoll C., Jorgensen K., Motawia M.S., Olsen C.E., Dixon D.P., Edwards R., Møller B.L. (2018). Glutathione transferases catalyze recycling of auto-toxic cyanogenic glucosides in sorghum. Plant J. Cell Mol. Biol..

[56] Yachdav G., Kloppmann E., Kajan L., Hecht M., Goldberg T., Hamp T., Hönigschmid P., Schafferhans A., Roos M., Bernhofer M. (2014). PredictProtein—An open resource for online prediction of protein structural and functional features. Nucleic Acids Res..

[57] Jensen K., Osmani S.A., Hamann T., Naur P., Møller B.L. (2011). Homology modeling of the three membrane proteins of the dhurrin metabolon: Catalytic sites, membrane surface association and protein–protein interactions. Phytochemistry.

[58] Vazquez-Albacete D., Montefiori M., Kol S., Motawia M.S., Møller B.L., Olsen L., Nørholm M.H.H. (2017). The CYP79A1 catalyzed conversion of tyrosine to (E)-p-hydroxyphenylacetaldoxime unravelled using an improved method for homology modeling. Phytochemistry.

[59] Hasemann C.A., Kurumbail R.G., Boddupalli S.S., Peterson J.A., Deisenhofer J. (1995). Structure and function of cytochromes P450:a comparative analysis of three crystal structures. Structure.

[60] Blomstedt C.K., Gleadow R.M., O’Donnell N., Naur P., Jensen K., Laursen T., Olsen C.E., Stuart P., Hamill J.D., Møller B.L. (2012). A combined biochemical screen and TILLING approach identifies mutations in *Sorghum bicolor* L. Moench resulting in acyanogenic forage production. Plant Biotechnol. J..

[61] Skelton J.L. (2014). EMS Induced Mutations in Dhurrin Metabolism and Their Impacts on Sorghum Growth and Development.

[62] Knoch E., Motawie M.S., Olsen C.E., Møller B.L., Lyngkjær M.F. (2016). Biosynthesis of the leucine derived α-, β- and γ-hydroxynitrile glucosides in barley (*Hordeum vulgare* L.). Plant J. Cell Mol. Biol..

[63] Kahn R., Fahrendorf T., Halkier B., Møller B. (1999). Substrate specificity of the cytochrome P450 enzymes CYP79A1 and CYP71E1 involved in the biosynthesis of the cyanogenic glucoside dhurrin in *Sorghum bicolor* (L.) Moench. Arch. Biochem. Biophys..

[64] Gleadow R.M., Veechies A.C., Woodrow I.E. (2003). Cyanogenic *Eucalyptus nobilis* is polymorphic for both prunasin and specific β-glucosidases. Phytochemistry.

[65] O’Donnell N.H., Møller B.L., Neale A.D., Hamill J.D., Blomstedt C.K., Gleadow R.M. (2013). Effects of PEG-induced osmotic stress on growth and dhurrin levels of forage sorghum. Plant Physiol. Biochem..

[66] Gleadow R.M., Ottman M.J., Kimball B.A., Wall G.W., Pinter P.J., LaMorte R.L., Leavitt S.W. (2016). Drought-induced changes in nitrogen partitioning between cyanide and nitrate in leaves and stems of sorghum grown at elevated CO_2_ are age dependent. Field Crops Res..

[67] Blomstedt C.K., Rosati V.C., Møller B.L., Gleadow R. (2018). Counting the costs: Nitrogen partitioning in *Sorghum* mutants. Funct. Plant Biol..

[68] Miller R.E., Gleadow R.M., Cavagnaro T.R. (2014). Age versus stage: Does ontogeny modify the effect of phosphorus and arbuscular mycorrhizas on above- and below-ground defence in forage sorghum?. Plant Cell Environ..

[69] Gleadow R.M., McKinley B.A., Blomstedt C.K., Lamb A.C., Møller B.L., Mullet J.E. (2021). Regulation of dhurrin pathway gene expression during *Sorghum bicolor* development. Planta.

[70] Bloom A.J., Chapin F.S., Mooney H.A. (1985). Resource Limitation in Plants-An Economic Analogy. Annu. Rev. Ecol. Syst..

[71] Wiedemuth K., Muller J., Kahlau A., Amme S., Mock H.P., Grzam A., Hell R., Egle K., Beschow H., Humbeck K. (2005). Successive maturation and senescence of individual leaves during barley whole plant ontogeny reveals temporal and spatial regulation of photosynthetic function in conjunction with C and N metabolism. J. Plant Physiol..

[72] Levin D.A. (1973). The role of trichomes in plant defense. Q. Rev. Biol..

[73] Tian D., Tooker J., Peiffer M., Chung S.H., Felton G.W. (2012). Role of trichomes in defense against herbivores: Comparison of herbivore response to woolly and hairless trichome mutants in tomato (*Solanum lycopersicum*). Planta.

[74] Johnson H.B. (1975). Plant pubescence: An ecological perspective. Bot. Rev..

[75] Pičmanová M., Neilson E.H., Motawia M.S., Olsen C.E., Agerbirk N., Gray C.J., Flitsch S., Meier S., Silvestro D., Jorgensen K. (2015). A recycling pathway for cyanogenic glycosides evidenced by the comparative metabolic profiling in three cyanogenic plant species. Biochem. J..

[76] Schmidt F.B., Cho S.K., Olsen C.E., Yang S.W., Møller B.L., Jørgensen K. (2018). Diurnal regulation of cyanogenic glucoside biosynthesis and endogenous turnover in cassava. Plant Direct.

[77] Goodger J.Q.D., Capon R.J., Woodrow I.E. (2002). Cyanogenic polymorphism in *Eucalyptus polyanthemos* Schauer subsp. *vestita* L. Johnson and K. Hill (Myrtaceae). Biochem. Syst. Ecol..

[78] Gleadow R.M., Haburjak J., Dunn J.E., Conn M.E., Conn E.E. (2008). Frequency and distribution of cyanogenic glycosides in *Eucalyptus* L’Herit. Phytochemistry.

[79] Gleadow R.M., Woodrow I.E. (2000). Polymorphism in cyanogenic glycoside content and cyanogenic β-glucosidase activity in natural populations of *Eucalyptus cladocalyx*. Aust. J. Plant Physiol..

[80] Armstrong H.E., Armstrong Edward F., Horton E. (1913). Herbage Studies. II. Variation in *Lotus corniculatus* and *Trifolium repens* (cyanophoric plants). Proc. R. Soc. Lond. Ser. B.

[81] Aikman K., Bergman D., Ebinger J., Seigler D. (1996). Variation of cyanogenesis in some plant species of the midwestern United States. Biochem. Syst. Ecol..

[82] Jones D.A. (1977). On the polymorphism of cyanogenesis in *Lotus corniculatus* L.. Heredity.

[83] Hughes M.A. (1991). The cyanogenic polymorphism in *Trifolium repens* L. (white clover). Heredity.

[84] Olsen K.M., Sutherland B.L., Small L.L. (2007). Molecular evolution of the *Li*/*li* chemical defence polymorphism in white clover (*Trifolium repens* L.). Mol. Ecol..

[85] Kakes P., Hakvoort H.W.J. (1994). On the origin of the cyanogenic polymorphism in *Trifolium repens* L.. J. Evol. Biol..

[86] Takos A.M., Knudsen C., Lai D., Kannangara R., Mikkelsen L., Motawia M.S., Olsen C.E., Sato S., Tabata S., Jørgensen K. (2011). Genomic clustering of cyanogenic glucoside biosynthetic genes aids their identification in Lotus japonicus and suggests the repeated evolution of this chemical defence pathway. Plant J. Cell Mol. Biol..

[87] Thodberg S., Del Cueto J., Mazzeo R., Pavan S., Lotti C., Dicenta F., Neilson E.H.J., Møller B.L., Sánchez-Pérez R. (2018). Elucidation of the amygdalin pathway reveals the metabolic basis of bitter and sweet almonds (*Prunus dulcis*). Plant Physiol..

[88] Brozynska M., Furtado A., Henry R.J. (2016). Genomics of crop wild relatives: Expanding the gene pool for crop improvement. Plant Biotechnol. J..

[89] Gleadow R., Bjarnholt N., Jørgensen K., Fox J., Miller R.M. (2012). Detection, identification and quantitative measurement of cyanogenic glycosides. Research Methods in Plant Science. Vol. 1. Soil Allelochemicals.

[90] Montini L., Crocoll C., Gleadow R.M., Motawia M.S., Janfelt C., Bjarnholt N. (2020). Matrix-Assisted Laser Desorption/Ionization-Mass Spectrometry Imaging of Metabolites during Sorghum Germination. Plant Physiol..

[91] Møller B.L., Olsen C.E., Motawia M.S. (2016). General and stereocontrolled approach to the chemical synthesis of naturally occurring cyanogenic glucosides. J. Nat. Prod..

[92] McLaren W., Gil L., Hunt S.E., Riat H.S., Ritchie G.R.S., Thormann A., Flicek P., Cunningham F. (2016). The ensembl variant effect predictor. Genome Biol..

[93] Allen G.C., Flores-Vergara M.A., Krasynanski S., Kumar S., Thompson W.F. (2006). A modified protocol for rapid DNA isolation from plant tissues using cetyltrimethylammonium bromide. Nat. Protoc..

[94] Halkier B.A., Møller B.L. (1989). Biosynthesis of the cyanogenic glucoside dhurrin in seedlings of *Sorghum bicolor* (L) Moench and partial purification of the enzyme system involved. Plant Physiol..

