# Supplementary material for: Cyanogenesis in the Sorghum Genus: From Genotype to Phenotype
Source: Genes (Basel). 2022 Jan 14;13(1):140. doi: 10.3390/genes13010140 (PMC8775130; doi:10.3390/genes13010140)
Supplement: Supplementary file 1 [file genes-13-00140-s001.zip › genes-1544261-supplementary.pdf]

genes

MDPI

Cyanogenesis in the *Sorghum* genus: from genotype to phenotype

Max Cowan [1,5], Birger Lindberg Møller [2], Sally Norton [3], Camilla Knudsen [2], Christoph Crocoll [2], Agnelo Furtado [4], Robert Henry [4], Cecilia Blomstedt [1] and Ros Gleadow [1,4]*

[1] School of Biological Sciences, Monash University, Clayton, Vic 3800 Australia;

[2] Plant Biochemistry Laboratory, Department of Plant and Environmental Sciences, University of Copenhagen, Denmark;

[3] Australian Grains Genebank, Agriculture Victoria, Horsham, Australia;

[4] Queensland Alliance for Agriculture and Food Innovation, The University of Queensland, St Lucia, QLD, Australia;

[5] Current address: Queensland Alliance for Agriculture and Food Innovation, The University of Queensland, Nambour, QLD, Australia max.cowan@uq.edu.au

Supplementary Figure S1 CLUSTAL O (1.2.4) multiple sequence alignment of the amino acid sequences of CYP79A1 obtained by PCR from wild sorghum species.

Table S1: Accession and provenance details of the sorghum crop wild relatives.

Table S2: Genes selected for variant analysis.

Table S3: Sequence of the primers used to amplify *CYP79A1* from the wild sorghum species.

Supplementary References

Supplementary Figure S1

CLUSTAL O (1.2.4) multiple sequence alignment of the amino acid sequences of CYP79A1 obtained by PCR from wild sorghum species.

Highlighted in blue are amino acids identified as important for function as determined using EMS mutant plants showing reduced HCNp. P414L and C493Y results in acyanogenic plants [1,2].

Yellow Highlighted regions are motifs shown to be important by modelling and experimental analysis [3,4]. E408, R411 and R460 form the E-R-R triad which is key to docking of the substrate tyrosine.

```
                                    Transmembrane region
brachypodum             MATKEVEAAAATVLAAPLLSTSTILKLLLFVVTLSYLARALSRPRKTTTKCSSS--A---  55
plumosum                MATNEVEAAAATVLAAPLLSTSTILKLLLSVVALSYLARALSRPRKTTTKCSS-TTS---  56
interjectum             MATMEVEAAAATVLAAPLLSTSTILKLLLFVVTLSYLARALSRPRKTTTKCGS-TTS---  56
amplum                  MATMEVEAAAATVLDAPLLSTSTILKLLLFVGTLSYLARALSRPRKTTTKCSS-TTS---  56
bulbosum                MATMEVEAAAATVLGAPLLSTSTILKLLLFVVTLSYLARALSRPRKTTTKCSSSTTS---  57
purpureosericeum        MATKEVEAAAATVLAAPLLSTSAILKLLLFVVTLSYLARALRRPRKTTTN-----KCS--  53
versicolor              MATMEVEAAAATVLAAPLLSTSAILKLLLFVVTLSYLARALSRPRKTTTKCSSTTSSG--  58
bicolor                 MATMEVEAAAATVLAAPLLSSSAILKLLLFVVTLSYLARALRRPRKSTTKCSSTTCASPP  60
laxiflorum              MATMEVDAAAATVLAAPLLSSSAILKLLLFVVTVSYLARALSRPRKTSTKCSSTTCASP-  59
angustum                MATMEVDAAGATVLAAPLLSSSAILKLLLVVATLSYLSRALIRPRKTTTKCSSTT-----  55
ecarinatum              MATMEVEAAAATALAPPLLSSSAILKLLLVVATLSYLSRALIRPRKTTTKCSSTT-----  55
intrans                 MATMEVEAAAATVLAAPLLSSSAILKLLLFVATLSYLSRALIRPRKTTTKCGSTT-----  55
                        *** **:**.**.* ****:*:****** * ::***:*** ****::*:

brachypodum             GVGSNPALPPGPVPWPIVGNLPEMLMNKPAFRWIHQMMREMGTDIACVKLGGIHVVSITC  115
plumosum                GVGSNPALPPGPVPWPIVGNLPEMVINKPAFRWIHQMMREMGTDIACVKLGGIHVVSITC  116
interjectum             CVGSNPPLPPGPVPWPIVGNLPEMLINKPAFRWIHQMMREMGTDIACVKLGGIHVVSITC  116
amplum                  CVGSNPALPPGPVPWPIVGNLPEMLINKPAFRWIHQMMREMGTDIACVKLGGIHVVSITC  116
bulbosum                CVGSNPALPPGPVPWPIVGNLPEMLINKPAFRWIHQMMREMGTDIACVKLGGIHVVSITC  117
purpureosericeum        -VGSNPPLPPGPVPWPIVGNLPEMLLNKPAFRWIHQMMRDMGTDIACVKLGGIHVVSITC  112
versicolor              -VGSNPPLPPGPVPWPVVGNLPEMLLNKPAFRWIHQIMRDMGTDIACVKLGGVHVVSITC  117
bicolor                 AGVGNPPLPPGPVPWPVVGNLPEMLLNKPAFRWIHQMMREMGTDIACVKLGGVHVVSITC  120
laxiflorum              -GVGNPPLPPGPVPWPVVGNLPEMLLNKPAFRWIHQMMHEMGTDIACVKLGGVHVVSITC  118
angustum                -GVGNLPLPPGPAPWPVVGNLPEMLLNKPAFRWIHQMMSEMGTGIACVKLGDVHVVCVTC  114
ecarinatum              -GVGNLPLPPGPAPWPVVGNLPEMLLNKPAFRWIHQMMSEMGTDMACVKLGDVHVVCVTC  114
intrans                 -GVGNLPLPPGPAPWPVVGNLPEMLLNKPAFRWIHQMMSEMGTDIACVKLGAVHVVCVTC  114
                           .*  *****.***:*******::**********:* :***.:****** :***.:**

                                               E145   R152       Heme binding
brachypodum             PEMAREVLRKQDANFISRPLTFASETFSGGYRNAVLSPYGDQWKKMRRVLTSEIICPSRH  175
plumosum                PEIAREVLRKQDANFISRPLTFASETFSGGYRNAVLSPYGDQWKKMRRVLTSEIICPSRH  176
interjectum             PEMAREVLRKQDANFVSRPLTFASETFSGGYRNAVLSPYGDQWKKMRRVLTSEIICPSRH  176
amplum                  PEIAREVLRKQDANFTSRPLTFASETFSGGYRNAVLSPYGDQWKKMRRVLTSEIICPSRH  176
bulbosum                PEIAREVLRKQDANFISRPLTFASETFSGGYRNAVLSPYGDQWKKMRRVLTSEIICPSRH  177
```

```
purpureosericeum    PEIAREVLRKQDANFISRPLTFASETFSGGYRNAVLSPYGDQWKKMRRVLTSEIICPSRH 172
versicolor          PEIAREVLRKQDANFISRPLTFASETFSGGYRNAVLSPYGDQWKKMRRVLTSEIICPSRH 177
bicolor             PEIAREVLRKQDANFISRPLTFASETFSGGYRNAVLSPYGDQWKKMRRVLTSEIICPSRH 180
laxiflorum          PEIAREVLRKQDANFISRPLTFASEMFSGGYRNAVLSPYGDQWKKMRRVLTSEIICPSRH 178
angustum            PEIAREVLRKQDANFISRPLTFASETFSGGYRNAVLSPYGAQWKKMRRVLTSEIICPPRH 174
ecarinatum          PEIAREVLRKQDANFISRPLTFASETFSGGYRNAVLSPYGAQWKKMRRVLTSEIICPSRH 174
intrans             PEIAREVLRKQDANFISRPLTFASETFSGGYRNAVLSPYGAQWKKMRRVLTSEIICPSRH 174
                    **:************ ********* ************** **************** **

brachypodum         AWLHDKRADEADNLTRYVYNLATKAA-AGDDAVVDVRHVARHYCGNVIRRLMFNRRYFGE 234
plumosum            AWLHDKRADEADNLTRYVYNLATKAAAAAGDDVVDVRHVARHYCGNVIRRLMFNRRYFGE 236
interjectum         AWLHDKRADEADNLTRYVYNLATKAA-AGDDVVVDVRHVARHYCGNVIRRLMFNRRYLGE 235
amplum              AWLHDKRADEADNLTRYVYNLATKAA-AAAGDVVDVRHAARHYCGNVIRRLMFNRRYFGE 235
bulbosum            AWLHDKRADEADNLTRYVYNLATKAA-AAGDDVVDVRHVARHYCGNVIRRLMFNRRYFGE 236
purpureosericeum    AWLHDKRTDEADNLTRYVYNLATTKA-TGRDDVVDVRHVARHYCGNVIRRLMFNRRYFGE 231
versicolor          AWLHDKRTDEADNLTRYVYNLATTKA-TGG-DVVDVRHVARHYCGNVIRRLMFNKRYFGE 235
bicolor             AWLHDKRTDEADNLTRYVYNLATKAA-T-GDVAVDVRHVARHYCGNVIRRLMFNRRYFGE 238
laxiflorum          AWLHDKRTDEADNLTRYIYNLATKSA-G---DVVDVRHVARHYCGNVIRRLMFNRRYFGE 234
angustum            VWLHDKRSDEADNLTRYVYNLAT-GA--AGGNVVDVRHIARHYCGNVIRRLMFNKRYFGE 231
ecarinatum          VWLHDKRADEADNLTRYVYNLATTRA--AGGNVVDVRHIARHYCGNVIRRLMFNKRYFGE 232
intrans             AWLHDKRADEADNLTRYVYNLATTRA--AGGNVVDVRHIARHYCGNVIRRLMFNKRYFGE 232
                    .******:*********:*****  *      .***** ***************:**:**

brachypodum         PQPDGGPGPMEELHMDAVFTSLGLLYAFCVSDYLPWLRGLDLDGHEKIVKEANQTVNRLH 294
plumosum            PQPDGGPGPMEELHMDAVFTSLGLLYAFCVSDYLPWLRGLDLDGHEKIVKQANETVNRLH 296
interjectum         PQPDGGPGPMEELHMDAVFTSLGLLYAFCVSDYLPWLRGLDLDGHEKIVKEANETVNRLH 295
amplum              PQPDGGPGPMEELHMDAVFTSLGLLYAFCVSDYLPWLRGLDLDGHEKIVKEANETVNRLH 295
bulbosum            PQPDGGPGPTEELHMDAVFTSLGLLYAFCVSDYLPWLRGLDLDGHERIVKEANETVNRLH 296
purpureosericeum    PQPDGGPGPMEELHMDAVFTSLGLLYAFCVSDYLPWLRGLDLDGHEKIVKEANETVNRLH 291
versicolor          PQPDGGPGPMEVLHMDAVFTSLGLLYAFCVSDYLPWLRGLDLDGHEKIVKEANAAVNRLH 295
bicolor             PQADGGPGPMEVLHMDAVFTSLGLLYAFCVSDYLPWLRGLDLDGHEKIVKEANVAVNRLH 298
laxiflorum          PQPDGGPGPMEVLHMDAVFTSLGLLYAFCVSDYLPWLRGLDLDGHEKIVKEANEAVNRLH 294
angustum            PQPDGGPGPMEVLHMDAVFTSLGLLYAFCISDYLPWLRGLDLDGHERMVKEANETVNRLH 291
ecarinatum          PQPDGGPGPMEVLHMDAVFTSLGLLYAFCISDYLPWLRGLDLDGHEKIVKEANETVNRLH 292
intrans             PQPDGGPGPMEVLHMDAVFTSLGFLYAFCISDYLPWLRGLDLDGHEKIVKEANETVNRLH 292
                    ** ****** * ***********:*****:****************::**:** :*****

                                                                D347   D354 N355
brachypodum         DTVIDGRWRQWKSGERKEMEDFLDVLITLKDAQGNPLLTIEEVKAQSQDITFAAVDNPSN 354
plumosum            DAVIDDRWRQWKSGERKEMEDFLDVLITLKDAQGNPLLTIEEVKAQSQDITFAAVDNPSN 356
interjectum         DAVIDDRWRQWKSGERKEMEDFLDVLITLKMAQGNPLLTIEEVKAQSQDITFAAVDNPSN 355
amplum              DAVIDDRWRQWKSGERKEMEDFLDVLITLKDAQGNPLLTIEEVKAQSQDITFAAVDNPSN 355
bulbosum            DTVIDDRWRQWKSGERKEMEDFLDVLITLKDAQGNPLLTIEEVKAQSQDITFAAVDNPSN 356
```

```
purpureosericeum   DTVIDDRWRQWKSGERQEMEDFLDVLITLKDAQGNPLLTIEEVKAQSQDITFAAVDNPSN 351
versicolor         DTVIDARWRQWKSGERKEMEDFLDVLITLKDAQGNPLLTIEEVKAQSQDITFAAVDNPSN 355
bicolor            DTVIDDRWRQWKSGERQEMEDFLDVLITLKDAQGNPLLTIEEVKAQSQDITFAAVDNPSN 358
laxiflorum         DTVIDARWRQWKSGERQEMEDFLDVLITLKDAQGNPLLTIEEVKAQSQDITFAAVDNPSN 354
angustum           DMVIDDRWRRWKSGERQEMEDFLDVLITFMDTEGNPLLAIEEVKAQLQDITFAAVDNPSN 351
ecarinatum         DMVIDDRWRRWKSGERQEMEDFLDVLITLKDTEGSPLLTIEEVKAQLQDITFAAVDNPSN 352
intrans            DMVIDDRWRRWKSGQRQEIQDFLDVLITLKDTEGNPLLTIEEVKAQLQDITFAAVDNPSN 352
                   * *** ***:****:*:*::********:  ::*.***:******* ************
```

E408 R411 P414

```
brachypodum        AVEWALAEMVNNPEVMAKAMEELDRVVGRDRLVQESDIPKLNYVKACIREAFRLHPVAPF 414
plumosum           AVEWALAEMVNNPEVMAKATEELDRVVGRDRLVQESDIPKLNYVKACIREAFRLHPVAPF 416
interjectum        AVEWALAEMVNNPEVMAKATEELDRVVGRDRLVQESDIPKLNYVKACIREAFRLHPVAPF 415
amplum             AVEWALAEMVNNPEVMAKAMEELDRVVGRDRLVQESDIPKLNYVKACIREAFRLHPVAPF 415
bulbosum           AVEWALAEMVNNPEVMAKAMEELDRVVGRDRLVQESDIPKLNYVKACIREAFRLHPVAPF 416
purpureosericeum   AVEWALAEMVNKPEVMAKAMEELDRVVGRERLVQESDIPKLNYVKACIREAFRLHPVAPF 411
versicolor         AVEWALAEMVNNPEVMAKAMEELDRVVGRERLVQESDIPKLNYVKACIREAFRLHPVAPF 415
bicolor            AVEWALAEMVNNPEVMAKAMEELDRVVGRERLVQESDIPKLNYVKACIREAFRLHPVAPF 418
laxiflorum         AVEWALAEMVNNPEVMTKATEELDRVVGRERLVQESDIPKLNYVKACIREAFRLHPVAPF 414
angustum           AAEWAVAEMVNNLEVMAKAMEELDRVVGRQRLVQESDIPKLNYVKACIREAFRLHPVAPF 411
ecarinatum         AAEWALAEMVNNPEVMAKAMEELDRVVGRQRLVQESDIPKLNYVKACIREAFRLHPVAPF 412
intrans            AAEWALAEMVNNPEVMAKAMEELDRVVGRQRLVQESDIPKLNYVKACIREAFRLHPVAPF 412
                   *.***:*****: ***:** *********:******************************
```

PERF domain - R460

```
brachypodum        NVPHVALDDTTIAGYRVPKGSHVILSRTGLGRNPRVWDEPLRFYPDRHLAAA-ADVVLTE 473
plumosum           NVPHVALDDTTIAGYRVPKGSHVILSRTGLGRNPRVWDEPLRFYPDRHLAAA-ADVVLTE 475
interjectum        NVPHVALDDTTIAGYRVPKGSHVILSRTGVGRNPRVWDEPLRFYPDRHLAAA-TDVVLTE 474
amplum             NVPHVALDDTTIAGYRVPKGSHVILSRTGLGRNPRVWDEPLRFYPDRHLAAA-TDVVLTE 474
bulbosum           NVPHVALDDTTIAGYRVPKGSHVILSRTGLGRNPRVWDEPLRFYPDRHLAAA-TDVVLTE 475
purpureosericeum   NVPHVALADTTIAGYHVPKGSHVILSRTGLGRNSRVWDEPLRFYPDRHLAA--ADVVLTE 469
versicolor         NVPHVALADTTIAGYHVPKGSHVILSRTGLGRNSRVWDEPLRFYPDRHLAA--ADVVLTE 473
bicolor            NVPHVALADTTIAGYRVPKGSHVILSRTGLGRNPRVWDEPLRFYPDRHLATAASDVALTE 478
laxiflorum         NVPHVALADTTIAGYRVPKGSHVILSRTGLGRNPRVWDEPLRFYPDRHLAA-ASDVALTE 473
angustum           NVPRVALADTTIAGCRVPKDSHLILSRTGLGRNPRVWEEPLRFYPYISITSV-SDVGRSG 470
ecarinatum         NVPRVALADTTIAGCRVPKGSHVILSRTGLGRNPRVWEEPLRFYPDRHLASV-SDVALTE 471
intrans            NVPRVALADTTIAGCRLPKDSHVILSRTGLGRNPRVWEEPLRFYPDRHLASV-SDVALTE 471
                   ***:*** ****** ::**.**:******:*** ***:*******   :::  :**  :
```

```
                                       C493
brachypodum         NDLRFISFSTGRRGCIAASLGTAMNIMLFGRLLQGFTWSKPAGVEAVDLSESKSDTFMAT  533
plumosum            NDLRFISFSTGRRGCIAASLGTAMSIMLFGRLLQGFTWSKPAGVEAVDLSESKSDTFMAT  535
interjectum         NDLRFISFSTGRRGCIAASLGTAMSIMLFGRLLQGFTWSKPAGVEAVDLSESKSDTFMAT  534
amplum              NDLRFISFSTGRRGCIAASLGTAMSIMLFSRLLQGFTWSKPAGVEAVDLSESKSDTFMAT  534
bulbosum            NDLRFISFSTGRRGCIAASLGTAMSIMLFGRLLQGFTWSKPAGVEAVDLSESKSDTFMAT  535
purpureosericeum    NDLRFISFSTGRRGCIAASLGTAMSIMLFGRLLQGFAWSKPAGVEAVDLSESKSDTFMAT  529
versicolor          NDLRFISFSTGRRGCIAASLGTAMSIMLFGRLLQGFTWSKPAGVEAVDLSESKSDTFMAT  533
bicolor             NDLRFISFSTGRRGCIAASLGTAMSVMLFGRLLQGFTWSKPAGVEAVDLSESKSDTFMAT  538
laxiflorum          NDLRFISFSTGRRGCIAASLGTAMSIMLFGRLLQGFTWSKPAGVEAVDLSESKSDTFMAT  533
angustum            KDLRFISFSTGGRGCIAASLGTTMSIMLFGRLLQGFTWSKPAGVEAVDLSECKNTTFMAT  530
ecarinatum          NDLRFISFSTGRRGCIAASLGTAMSIMLFGRLLQGFTWSKPAGVEAVDLSESKNDTFMAT  531
intrans             NDLRFISFSTGRRGCIAASLGTTMSIMLFGRLLQGFTWSKPAGVEAVDLSESKNGTFMAT  531
                    :********** **********:*.:***.******:**************.*. *****

brachypodum         PLVLHAEPRLPAHLYPTISV--- 553
plumosum            PLALHAEPRLPAHLYPSISI--- 555
interjectum         PLVLHAQPRLPAHLYPSISI--- 554
amplum              PLVLHAEPRLPAHLYPSFSI--- 554
bulbosum            PLVLHAEPRLPAHLYPSISSI-- 556
purpureosericeum    PLVLRAEPRLPAHLYPSFSI--- 549
versicolor          PLVLRAEPRLPARLYPSISI--- 553
bicolor             PLVLHAEPRLPAHLYPSISI--- 558
laxiflorum          PLVLNAEPRLPAHLYPAISI--- 553
angustum            PLALNAVPRLPVHLYPSISI--- 550
ecarinatum          PLALNAEPRLLVHLYPSFSI--- 551
intrans             PLALNAEPRLPVHLYPAISPPSD 554
                    **.*.* *** .:***::*
```

Table S1: Accession and provenance details of the sorghum crop wild relatives examined in the current study. Seeds were obtained from the Australian Grains Genebank (AGG), Horsham, Victoria (Coordinator: Dr Sally Norton). *denotes species of the *Eusorghum* subgenus.

Species	Accession number	Provenance	Latitude	Longitude
**HCN analysis**				
S. amplum	302623	Kimberley, WA	-14.5982	125.7928
S. angustum	302596	Central Highlands, QLD	-13.4583	142.9613
S. brachypodum	302670	Kakadu, NT	-12.5667	-132.8833
S. bulbosum	302645	Kimberley, WA	-16.0965	128.39
S. ecarinatum	302656	Kimberley, WA	-17.1838	124.9158
S. exstans	302577	Melville Island, NT	-11.6403	130.6317
S. interjectum	302428	Kimberley, WA	-15.7695	128.6462
S. intrans	302394	Katherine, NT	-14.4503	132.2432
S. laxiflorum	302525	Katherine, NT	-16.6477	135.8495
S. leiocladum	300170	New England, NSW	-28.9263	152.3453
S. macrospermum	302367	Katherine, NT	-14.4097	132.1977
S. matarankense	302521	Katherine, NT	-16.0797	136.3077
S. plumosum	302415	Katherine, NT	-14.7922	131.9427
*S. propinquum**	302546	NA	NA	NA
S. purpureosericeum	318068	NA	NA	NA
S. stipoideum	302442	Kimberley, WA	-18.0412	127.8037
S. timorense	302660	Kimberley, WA	-17.3657	124.2937
S. versicolor	321128	Usagara, Tanzania	NA	NA
*S. halepense**	300167	Western Downs, QLD	-28	152
*S. × almum**	316842	Central Highlands, QLD	-24.1187	148.0901
**Illumina sequencing**				
S. brachypodum	302481	Arnhem Land, NT	-12.6767	132.8435
S. laxiflorum	302525	NT	-16.6477	135.8495
S. leiocladum	300148	Maidenwell, QLD	-26	151
S. macrospermum	302367	Katherine, NT	-14.4097	132.1977
S. matarankense	302521	Booraloola, NT	-16.0797	136.3077
S. purpureosericeum	318068	NA	NA	NA
**PCR**				
S. amplum	302455	WA	-15.977	128.9632
S. angustum	302588	QLD	-15.308	144.8133
S. brachypodum	302480	Arnhem Land, NT	-12.7145	132.4167
S. bulbosum	302417	Katherine, NT	-15.1425	131.6812
S. ecarinatum	302581	Katherine, NT	-14.4393	132.2755
S. exstans	302401	Katherine, NT	-14.553	133.0117
S. interjectum	302569	Nhulunbuy, NT	-12.7758	136.23
S. intrans	302389	NT	-13.5838	131.4568
S. laxiflorum	302503	QLD	-17.8917	139.2853
S. leiocladum	300148	Maidenwell, QLD	-26	151
S. macrospermum	322277	Katherine, NT	NA	NA
S. matarankense	302521	Booraloola, NT	-16.0797	136.3077
S. plumosum	322440	QLD	-20.8777	144.51245
S. purpureosericeum	321133	NA	NA	NA
S. stipoideum	302449	Kimberley, WA	-18.0043	125.2893
S. timorense	302634	Cloncurry, QLD	-20.6457	141.0265
S. versicolor	321127	NA	NA	NA

Table S2: Genes selected for variant analysis. Enzyme family, identification, function, and reference information for the genes examined in this study involved in dhurrin biosynthesis, bioactivation, recycling and transport. In addition, two genes were selected that are putatively involved in ethylene synthesis and tyrosine biosynthesis.

Enzyme	Gene	Gene ID	Function	Reference
Cytochrome P450	*CYP79A1*	Sobic.001G012300	Biosynthesis	[5]
Cytochrome P450	*CYP71E1*	Sobic.001G012200		[5]
UDP-glucosyltransferase	*UGT85B1*	Sobic.001G012400		[5]
Cytochrome P450 reductase	*POR*	Sobic.002G295100		[5]
MATE transporter	*MATE*	Sobic.001G012600	Transport	[6]
α-hydroxynitrile lyase	*HNL*	Sobic.004G335500	Bioactivation	[5]
β-glucosidase	*DHR1*	Sobic.008G079800		[7]
β-glucosidase	*DHR2*	Sobic.008G080400		[7]
β-cyanoalanine synthase	*BCAS*	Sobic.006G016900	HCN detoxification	[5]
β-cyanoalanine synthase	*CAS26*	Sobic.003G333700		[8]
Nitrilase	*NIT4A*	Sobic.004G225200	HCN detoxification, N recycling	[5]
Nitrilase	*NIT4B1*	Sobic.004G225000		[5]
Nitrilase	*NIT4B2*	Sobic.004G225100		[5]
Glutathione S-transferase	*GST1*	Sobic.001G012500	N recycling	[5,7,9]
Glutathione S-transferase	*GST1B*	Sobic.001G065800		[5]
Glutathione S-transferase	*GST3*	Sobic.003G416300		NA
ACC oxidase	*ACC*	Sobic.003G197200	Ethylene synthesis	
Chorismate mutase	*CM7*	Sobic.007G141500	Tyrosine synthesis	NA

Table S3: Details of the primers used to amplify and sequence *CYP79A1* from the wild sorghum species.

Primer name	Sequence	Tm (°C)	Application
CYP79A1 coding For	5′ GGCGACAATGGAGGTAGAGG 3′	66	PCR and
CYP79A1 coding Rev	5′ GATGGAGATGGACGGGTAGAG 3′	65	sequencing
CYP79A1 Int Rev 5′ 1	5′ ATTAATTACCTGTGACTGCGC 3′	61	sequencing
CYP79A1 Int Rev 5′ 2	5′ CGCATCTTCTTCCACTGGTC 3′	65	sequencing
CYP79A1 Int For 3′ 1	5′ CTTCCTGGATGTGCTCATCAC 3′	65	sequencing
CYP79A1 Int For 3′ 2	5′ GCGAAGGCGATGGAGGAG 3′	68	sequencing

Supplementary References

1. Blomstedt, C.K.; Gleadow, R.M.; O'Donnell, N.; Naur, P.; Jensen, K.; Laursen, T.; Olsen, C.E.; Stuart, P.; Hamill, J.D.; Møller, B.L., et al. A combined biochemical screen and TILLING approach identifies mutations in *Sorghum bicolor* L. Moench resulting in acyanogenic forage production. *Plant Biotechnology Journal* **2012**, *10*, 54-66, doi:10.1111/j.1467-7652.2011.00646.x.
2. Skelton, J.L. EMS induced mutations in dhurrin metabolism and their impacts on sorghum growth and development. Purdue University, West Lafayette, Indiana, 2014.

3. Hasemann, C.A.; Kurumbail, R.G.; Boddupalli, S.S.; Peterson, J.A.; Deisenhofer, J. Structure and function of cytochromes P450:a comparative analysis of three crystal structures. *Structure* **1995**, *3*, 41-62.
4. Vazquez-Albacete, D.; Montefiori, M.; Kol, S.; Motawia, M.S.; Møller, B.L.; Olsen, L.; Nørholm, M.H.H. The CYP79A1 catalyzed conversion of tyrosine to (E)-p-hydroxyphenylacetaldoxime unravelled using an improved method for homology modeling. *Phytochemistry* **2017**, *135*, 8-17, http://doi.org/10.1016/j.phytochem.2016.11.013.
5. Nielsen, L.J.; Stuart, P.; Pičmanová, M.; Rasmussen, S.; Olsen, C.E.; Harholt, J.; Møller, B.L.; Bjarnholt, N. Dhurrin metabolism in the developing grain of Sorghum bicolor (L.) Moench investigated by metabolite profiling and novel clustering analyses of time-resolved transcriptomic data. *BMC Genomics* **2016**, *17*, 1021.
6. Darbani, B.; Motawia, M.S.; Olsen, C.E.; Nour-Eldin, H.H.; Møller, B.L.; Rook, F. The biosynthetic gene cluster for the cyanogenic glucoside dhurrin in *Sorghum bicolor* contains its co-expressed vacuolar MATE transporter. *Scientific reports* **2016**, *6*, 37079, doi:10.1038/srep37079.
7. Hayes, C.M.; Burow, G.B.; Brown, P.J.; Thurber, C.; Xin, Z.; Burke, J.J. Natural Variation in Synthesis and Catabolism Genes Influences Dhurrin Content in Sorghum. *The Plant Genome* **2015**, *8*, doi:10.3835/plantgenome2014.09.0048.
8. Akbudak, M.A.; Filiz, E.; Uylas, S. Identification of O-acetylserine(thiol)lyase (OASTL) genes in sorghum (*Sorghum bicolor*) and gene expression analysis under cadmium stress. *Molecular biology reports* **2019**, *46*, 343-354, doi:10.1007/s11033-018-4477-0.
9. Bjarnholt, N.; Neilson, E.H.J.; Crocoll, C.; Jorgensen, K.; Motawia, M.S.; Olsen, C.E.; Dixon, D.P.; Edwards, R.; Møller, B.L. Glutathione transferases catalyze recycling of auto-toxic cyanogenic glucosides in sorghum. *The Plant journal : for cell and molecular biology* **2018**, *94*, 1109-1125, doi:10.1111/tpj.13923.
